# Understanding the Impact of Different Doses of Reducose^®^ Mulberry Leaf Extract on Blood Glucose and Insulin Responses after Eating a Complex Meal: Results from a Double-Blind, Randomised, Crossover Trial

**DOI:** 10.3390/nu16111670

**Published:** 2024-05-29

**Authors:** Pariyarath Sangeetha Thondre, Isabel Butler, Jonathan Tammam, Ifunanya Achebe, Elysia Young, Michael Lane, Andrew Gallagher

**Affiliations:** 1 Oxford Brookes Centre for Nutrition and Health, Oxford OX3 0BP, UK; pthondre@brookes.ac.uk (P.S.T.); isabel.hatami@paediatrics.ox.ac.uk (I.B.); jtammam@brookes.ac.uk (J.T.); ifyachebe21@gmail.com (I.A.); young.elysia@yahoo.co.uk (E.Y.); 2 Department of Paediatrics, University of Oxford, Oxford OX3 9DU, UK; 3 Phynova Group Limited, Banbury OX16 3ED, UK; mlane@phynova.com

**Keywords:** mulberry leaf extract, glycaemic response, insulinemic response, 1-deoxynojirimycin

## Abstract

Non-communicable diseases (NCDs) are becoming an increasingly important health concern due to a rapidly ageing global population. The fastest growing NCD, type 2 diabetes mellitus (T2DM), is responsible for over 2 million deaths annually. Lifestyle changes, including dietary changes to low glycemic response (GR) foods, have been shown to reduce the risk of developing T2DM. The aim of this study was to investigate whether three different doses of Reducose^®^, a mulberry leaf extract, could lower the GR and insulinemic responses (IR) to a full meal challenge in healthy individuals. A double-blind, randomised, placebo-controlled, repeat-measure, crossover design trial was conducted by the Oxford Brookes Centre for Nutrition and Health; 37 healthy individuals completed the study. Participants consumed capsules containing either 200 mg, 225 mg, 250 mg Reducose^®^ or placebo before a test meal consisting of 150 g white bread and egg mayo filler. Capillary blood samples were collected at 15-min intervals in the first hour and at 30-min intervals over the second and third hours to determine glucose and plasma insulin levels. The consumption of all three doses of Reducose^®^ resulted in significantly lower blood glucose and plasma insulin levels compared to placebo. All three doses of Reducose^®^ (200 mg, 225 mg, 250 mg) significantly lowered glucose iAUC 120 by 30% (*p* = 0.003), 33% (*p* = 0.001) and 32% (*p* = 0.002), respectively, compared with placebo. All three doses of Reducose^®^ (200 mg, 225 mg, 250 mg) significantly lowered the plasma insulin iAUC 120 by 31% (*p* = 0.024), 34% (*p* = 0.004) and 38% (*p* < 0.001), respectively. The study demonstrates that the recommended dose (250 mg) and two lower doses (200 mg, 225 mg) of Reducose^®^ can be used to help lower the GR and IR of a full meal containing carbohydrates, fats and proteins.

## 1. Introduction

Annually, over 41 million people die due to complications from non-communicable diseases (NCDs) [1]. The four most prevalent NCDs are cardiovascular disease, cancers, chronic respiratory diseases and diabetes, which account for over 80% of all NCD-related deaths [1]. It is becoming increasingly important to control and prevent the proliferation of NCDs due to an ageing global population, which is predicted to create a substantial burden on healthcare systems and out-of-pocket spending [2]. However, the most prominent NCDs have been shown to be preventable through behaviour changes that reduce risk factors such as increasing exercise, improving diet, and reducing the consumption of harmful substances (e.g., nicotine, alcohol) [3].

Type-2 diabetes mellitus (T2DM) is one of the most common NCDs and is responsible for around 2 million deaths globally [1]. It is also one of the fastest-growing NCDs, as the prevalence of T2DM has quadrupled in the last three decades. T2DM can increase the risk of further health complications, the most common of these being cardiovascular disease, which is a leading cause of morbidity in T2DM patients [4].

One of the most impactful actions to improve long-term health and reduce complications in T2DM is to control postprandial glucose excursions [5]. Glucose excursions are known to increase oxidative stress in microvascular tissue and increase the risk of cardiovascular mortality, and this risk is independent of diabetic status and is continuous [6,7,8,9].

The type of food we eat has a direct impact on blood glucose excursions—consuming foods with a high glycaemic index (GI) and glycaemic load (GL) increases blood glucose responses. High GI/GL foods are associated with an increased incidence of diabetes [10,11] and an increased incidence of coronary heart disease [12]. Fortunately, studies have shown that changing to a low-GI diet can improve glycaemic control and lower cardiometabolic risk factors [13].

There are many factors that determine the glycaemic responses (GR) of carbohydrate-containing foods, such as cooking methods, processing and other ingredients consumed within a complete meal (e.g., fats, protein, fibre, etc.) [14] due to the interaction between nutrients, which can slow the rate of carbohydrate absorption and lower rise in blood glucose responses [15]. Low GR foods may have an important role to play in the prevention and control of those at risk of developing T2DM, and they have been shown to significantly reduce the frequency of hyperglycemia [16], decrease total cholesterol [17] and improve insulin sensitivity and postprandial insulin response (IR) [18]. Insulin secretion is primarily elicited by the carbohydrate content of food; however, other dietary compounds, such as amino acids and fatty acids, as well as gastrointestinal hormones, have been shown to have an insulinotropic effect [19].

Dietary supplements have also been shown to have an acute effect on postprandial GR and IR. Extracts from the leaves of *Morus alba* L. (white mulberry) have a long history of use in traditional medicine [20]. *M. alba* leaves contain various iminosugars, the most prominent of which is the piperidine iminosugar 1-deoxynojirimycin (DNJ), which has a D-glucose analogue structure with a nitrogen group replacing the oxygen on the pyranose ring [21]. Due to the structural similarity with carbohydrate monosaccharides, DNJ competitively inhibits carbohydrate enzymes such as α-glycosidase and amylase [21]. Reducose^®^, a proprietary food ingredient formed through a water-based extraction of *M. alba* leaves standardised to DNJ, has been shown across several clinical studies to significantly reduce the postprandial GR and IR compared to placebo following starch and sugar challenges [22,23].

The primary objective of this study was to determine the effect of Reducose^®^ compared with placebo in reducing the GR and IR in real-life scenarios, such as when taken alongside a complete meal with standardised amounts of carbohydrate, fat, protein and fibre. Previous studies have only investigated the effects of co-administration with simple carbohydrates; however, the co-consumption with other macronutrients may impact the rate of gastric emptying and have an insulinotropic effect. The secondary aim was to compare the GR and IR to different doses of Reducose^®^ (200 mg, 225 mg, 250 mg) with a matched placebo when taken at the start of a complete meal.

## 2. Materials and Methods

### 2.1. Study Design and Setting

A double-blind, placebo-controlled, randomised, repeat measure, crossover design trial was used to compare the glycaemic response (GR) and the insulinemic response (IR) of three different doses of a proprietary mulberry leaf extract, Reducose^®^, to a matched placebo when taken at the start of a meal. Participants acted as their own controls. The trial was conducted by Oxford Brookes Centre for Nutrition and Health at Oxford Brookes University. Ethical approval for the study was obtained from the University Research and Ethics Committee (UREC) at Oxford Brookes University (UREC Registration No: 140806 for glycaemic response and UREC Registration No: 110594 for insulinemic response). Participants were given full details of the study protocol and the opportunity to ask questions before giving written informed consent prior to participation. The study was retrospectively registered with a clinical trial registry, registration number ISRCTN18212231. The study was conducted between October 2019 and June 2021 (prolonged recruitment due to COVID-19 lockdowns in the UK).

### 2.2. Participants

Participants were recruited from the local community, including Oxford Brookes University staff and students. Prior to recruitment, all potential participants completed a health questionnaire. Participants were excluded from the trial if they met any of the following criteria: [a] Aged < 18 or >60 years, [b] pregnant or lactating, [c] Body Mass Index (BMI) < 18.5 or >30 kg/m^2^, [d] fasting blood glucose value > 6.1 mmol/L, [e] any known food allergy or intolerance, medical condition or using medication which is known to affect glucose regulation or appetite and/or influence digestion and absorption of nutrients, [f] known history of diabetes mellitus or the use of antihyperglycemic drugs or insulin to treat diabetes and related conditions, [g] known IBS, severe liver, heart or kidney problems or blood clotting disorders, [h] major medical or surgical event requiring hospitalisation in the last 3 months, [i] use of steroids, protease inhibitors or antipsychotics, [j] high vulnerability level for COVID-19, [k] displayed COVID-19 symptoms 14 days prior to any test session (high temperature, continuous cough, loss or change to sense of smell or taste).

### 2.3. Test Products and Test Meal

Three doses of Reducose^®^ dietary supplements containing 200 mg, 225 mg, or 250 mg mulberry leaf extract, standardised to contain 5% (*w*/*w*) DNJ (batch number 181102, manufactured by Hunan Hill Pharmaceutical Co. Ltd., Hunan, China) in capsule form were compared with a placebo capsule matched for size, weight and colour containing no mulberry leaf extract. All capsules had identical fill weights, with microcrystalline cellulose being added to compensate for different active doses. The Reducose^®^ dietary supplement and placebo capsules were provided by the study sponsor, Phynova Group Limited. The Reducose^®^ dietary supplement/placebo capsule was consumed with a small amount of water 10 min prior to the consumption of the test meal.

The test meal consisted of 150 g of Hovis^®^ White Bread Thick Slice and 50 g of Tesco^®^ Egg Mayo Filler, providing a macronutrient composition of 60.9% (68.7 g) carbohydrate, 16.6% (18.7 g) protein and 20.4% (10.2 g) fat. The test meal ingredients were weighed using a balance calibrated to UKAS/ISO standards and checked daily to ensure calibration had not drifted. All the test products/meals were stored in the test kitchen at Oxford Brookes Centre for Nutrition and Health, where the temperature is monitored and recorded daily. The Tesco Egg Mayo Filler was stored in a refrigerator. Water (250 mL) was served with the capsule and the meal.

### 2.4. Protocol

The study was conducted by Good Clinical Practice (GCP) certified researchers, and the protocol used was in accordance with ISO 26642 standards [24].

For all participants, anthropometric measurements were made in the fasted state during the first session. Height was recorded to the nearest centimetre using a stadiometer (Seca Ltd., Birmingham, UK), with participants standing erect and without shoes. Body weight was recorded to the nearest 0.1 kg, with participants wearing light clothing and no shoes. Body fat percentage was measured using a body composition analyser (Tanita BC-418 MA; Tanita UK Ltd., Yiewsley, UK). Muscle mass evaluation was not conducted during the study. A summary of physical characteristics is presented in Table 1.

Prior to the first day of testing, participants were asked to restrict their intake of alcohol and caffeine-containing drinks and to restrict their participation in intense physical activity (for example, long periods at the gym, excessive swimming, running, aerobics). Participants were also asked not to eat or drink after 9 pm the night before the test; however, water was allowed in moderation. In addition, participants were asked to consume the same food and drink and quantities the evening before each test. They were also asked not to change their diet or exercise routine during the study; however, food diaries were not maintained by the participants.

The supplements and placebo were tested once in random order on separate days, with at least a 48-h gap between measurements to minimise carry-over effects. There was a minimum of 10 days between Visit 1 and Visit 4 (including washout periods and the weekend closure of the centre). Tests began in the morning, before 10 am, following the participants’ 12-h overnight fast. Participants consumed the test meal at a comfortable pace within 10 min and remained sedentary during each session.

Blood samples were taken at −5 min and 0 min before consumption of the test meal and capsule, and the baseline was taken as the mean of the two values. Further blood samples were taken at 15, 30, 45, 60, 90, 120, 150 and 180 min after starting to eat the test meal. Blood was obtained by finger-prick using the Unitstik^®^3 single-use lancing device (Owen Mumford, Oxford, UK). Fingers were not squeezed to extract blood from the fingertip as this may dilute with plasma.

Blood glucose was measured using the HemoCue Glucose 201 DM analyser (HemoCue^®^ Ltd., Angelholm, Sweden), which was calibrated daily using a control solution from the manufacturer. For insulinemic response measurements, blood samples were collected into chilled microvette^®^ capillary collection tubes treated with Potassium EDTA (CB 300 K2E; Sarstedt Ltd., Nümbrecht, Germany). The microvette^®^ tubes were centrifuged, and 200 µL of the supernatant plasma was obtained. Insulin concentrations in the plasma samples were determined by electrochemiluminescence immunoassay using an automated analyser (Cobas^®^ E411; Roche Diagnostics, Basel, Switzerland).

### 2.5. Sample Size and Randomisation

Based on the results from a previous study investigating Reducose^®^ [23], it was estimated that the minimal detectable difference in means between the low-dose group and placebo would be a 25% reduction in the incremental area under curve for blood glucose. Using the within-patient standard deviation from Thondre et al. [23], it was estimated that a sample size of 31 participants would be required to achieve a power of 80% with type 1 error (α) set to 5% and 41 participants to achieve a power of 90%.

Researchers at Oxford Brookes Centre for Nutrition and Health who recruited the participants were unaware of the allocation sequence (concealed allocation). Participants were assigned to a study participant number according to the date that they were recruited into the study. The study participant number was used to identify the participants and their corresponding intervention sequence. The intervention sequence was generated by the study sponsor using an online random 4 × 4 Latin Square generator (twelve random 4 × 4 Latin Squares).

### 2.6. Statistical Analyses

Data were analysed using IBM Statistical Package for the Social Sciences (SPSS) version 22.0 (SPSS Inc., Chicago, IL, USA). Data are presented as mean, standard deviation (SD) and standard error of the mean (SEM) values. The blood glucose and plasma insulin iAUC for both Reducose^®^ and the placebo were calculated geometrically by applying the trapezoid rule according to the ISO standards [24]. Prior to statistical analysis, the normality of the data was tested using the Shapiro–Wilks statistic. A repeated measures ANOVA (for normally distributed data) and nonparametric Friedman test (where data were not normally distributed) were used to compare measurements between the following:

(1) Reducose^®^ 200 mg dietary supplement;
(2) Reducose^®^ 225 mg dietary supplement;
(3) Reducose^®^ 250 mg dietary supplement;
(4) Placebo.

Where there was an overall significant difference, post hoc analysis was performed with a Bonferroni correction. The primary outcome measures were Blood glucose concentrations, blood glucose iAUC (at 60, 90, 120, 150 and 180 min), peak blood glucose, and time of peak blood glucose. The secondary outcome measures were Plasma insulin concentrations, plasma insulin iAUC (at 60, 90, 120, 150 and 180 min), peak plasma insulin, and time of peak plasma insulin. Statistical significance was set at *p* < 0.05. No subset analyses were performed as the study was powered for whole group, not sub-group, analysis.

## 3. Results

### 3.1. Physical Characteristics

Forty-three healthy participants were recruited (18 male, 25 female; aged 18 to 56 years) for the study (Table 1). The age range for males was 18 to 56 years (Mean ± SD 27.8 ± 8.8 years), and the age range for females was 18 to 52 years (Mean ± SD 30.6 ± 10.8 years). Of the 43 participants recruited, four withdrew from the study (one moved away, two did not tolerate study procedures, and one did not give a reason), and two participants were excluded (one did not comply with the study procedures, and one was not eligible for the study). Therefore, the complete GR and IR data are reported for 37 participants who all completed the study. Of the 37 participants, the age range for females (*n* = 22) was 18 to 56 years (Mean ± SD 30.6 ± 10.8 years), and the age range for males (*n* = 15) was 19 to 52 years (Mean ± SD 28.6 ± 8.9 years). The physical characteristics of the included study population are presented in Table 1.

### 3.2. Glycaemic Response

**Figure 1:**
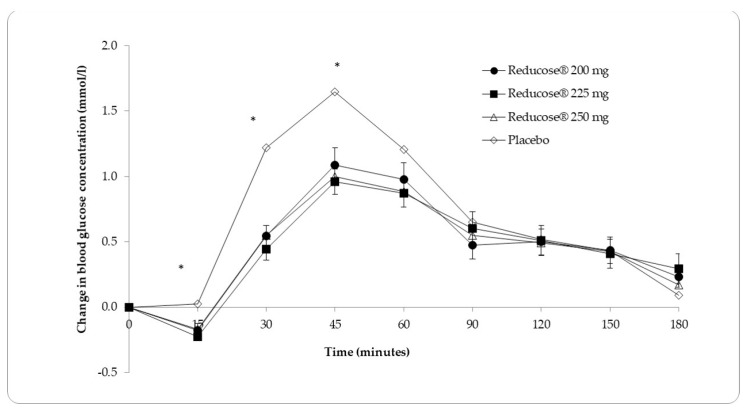
Mean (SEM) changes in blood glucose (mmol/L) for Reducose^®^ 200 mg, 225 mg and 250 mg dietary supplement compared to placebo (*n* = 37). * Statistically significant difference (*p* < 0.05) SEM, standard error of the mean.

Table 2 and Figure 1 present the mean blood glucose measurements and the mean change in blood glucose for the three doses of Reducose^®^ dietary supplement and placebo.

There was a significant overall difference in the actual blood glucose measurements between the placebo and the three doses of Reducose^®^ at 15 min (*F*(3, 108) = 6.366, *p* = 0.001), 30 min (*F*(3, 108) = 21.177, *p* < 0.001), 45 min *F*(2.413, 86.874) = 17.285, *p* < 0.001 and 60 min (*F*(3, 108) = 3.535, *p* = 0.017). Pairwise comparisons revealed significantly lower actual blood glucose measurements for Reducose^®^ 200 mg compared with placebo at 15 min (*p* = 0.039), 30 min (*p* < 0.001) and 45 min (*p* = 0.002). There were significantly lower blood glucose measurements for Reducose^®^ 225 mg at 15 min (*p* = 0.001), 30 min (*p* < 0.001), 45 min (*p* < 0.001) and 60 min (*p* = 0.034) compared to placebo. In the Reducose^®^ 250 mg group, blood glucose was significantly lower than placebo at 30 min (*p* < 0.001) and 45 min (*p* < 0.001). Between the placebo and three doses of Reducose^®^ groups, there was no significant change in actual blood glucose measurements at time points 90 min, 120 min, 150 min and 180 min. There were no significant differences in the glycaemic responses between the three doses of Reducose^®^.

Table 3 shows the mean glucose iAUC at 60, 90, 120, 150 and 180 min for the three doses of Reducose^®^ and Placebo. There was an overall significant difference in the glucose iAUC between the three doses of Reducose^®^ and placebo at 60 min (*F*(2.420, 87.138) = 20.550, *p* < 0.001), 90 min (*F*(3, 108) = 12.552, *p* < 0.001), 120 min (χ^2^(3) = 20.366, *p* < 0.001), 150 min (χ^2^(3) = 17.054, *p =* 0.001) and 180 min (χ^2^(3) = 12.677, *p =* 0.005). Post hoc analysis showed a significantly lower glucose iAUC for Reducose^®^ 200 mg compared to placebo at 60 min (*p* < 0.001), 90 min (*p* = 0.004), 120 min (*p* = 0.003), 150 min (*p* = 0.005) and 180 min (*p* = 0.036). In the 225 mg dose group, there was a significantly lower glucose iAUC compared with placebo at 60 min (*p* < 0.001), 90 min (*p* < 0.001), 120 min (*p* = 0.001), 150 min (*p* = 0.005) and 180 min (*p* = 0.011), and the 250 mg dose group exhibited an identical pattern with significantly lower glucose iAUCs at 60 min (*p* < 0.001), 90 min (*p* < 0.001), 120 min (*p* = 0.002), 150 min (*p* = 0.004) and 180 min (*p* = 0.031). All three doses of Reducose^®^ (200 mg, 225 mg and 250 mg) significantly lowered the primary endpoint, iAUC 120, by 30%, 33%, and 32% compared with placebo, respectively. There were no significant differences between the glucose iAUCs for the three doses of Reducose^®^.

There was an overall significant difference in the peak blood glucose (Cmax) between the three doses of Reducose^®^ and the placebo (χ^2^(3) = 27.808, *p* < 0.001). Pairwise comparisons demonstrated a significantly lower peak for Reducose^®^ 200 mg (*p* = 0.001), 225 mg (*p* < 0.001) and 250 mg (*p* = 0.002) compared to the placebo. None of the doses had a significant effect (χ^2^(3) = 5.554, *p =* 0.135) on the time to peak (Tmax) when compared with the placebo (Table 4).

### 3.3. Insulinaemic Response

**Figure 2:**
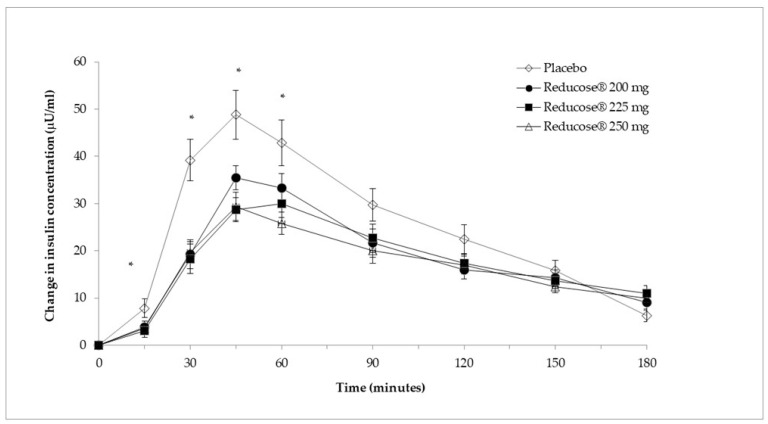
Mean (SEM) changes in plasma insulin (µU/mL) for Reducose^®^ 200 mg, 225 mg and 250 mg dietary supplement compared to placebo (*n* = 37) * Statistically significant difference (*p* < 0.05) SEM, standard error of the mean.

Table 5 and Figure 2 present the mean plasma insulin concentrations and the mean change in plasma insulin for the three doses of Reducose^®^ and placebo. There was an overall significant difference in the actual plasma insulin measurements between the three doses of Reducose^®^ and placebo at 15 min (χ^2^(3) = 10.07, *p =* 0.018), 30 min (χ^2^(3) = 29.724, *p <* 0.001), 45 min (χ^2^(3) = 27.324, *p <* 0.001) and 60 min (χ^2^(3) = 12.762, *p =* 0.005). Post hoc analysis showed significantly lower actual plasma insulin measurements for Reducose^®^ 200 mg compared to placebo at 30 min (*p* < 0.001) and 45 min (*p* = 0.013). There was a significant reduction in the actual plasma insulin measurements between Reducose^®^ 225 mg and placebo at 15 min (*p* = 0.018), 30 min (*p* < 0.001), 45 min (*p* < 0.001) and 60 min (*p* = 0.024). There were similar significant reductions in the actual plasma insulin measurements between Reducose^®^ 250 mg and placebo at 30 min (*p* = 0.002), 45 min (*p* < 0.001) and 60 min (*p* = 0.007).

Table 6 shows the mean plasma insulin iAUC at 60, 90, 120, 150 and 180 min for the three doses of Reducose^®^ and placebo. There was an overall significant difference in the insulin iAUC between the three doses of Reducose^®^ and placebo at 60 min (χ^2^(3) = 38.189, *p <* 0.001), 90 min (χ^2^(3) = 30.827, *p <* 0.001), 120 min (χ^2^(3) = 20.935, *p <* 0.001), 150 min (χ^2^(3) = 21.551, *p <* 0.001) and 180 min (χ^2^(3) = 18.762, *p <* 0.001). Pairwise comparisons showed a significant reduction in the insulin iAUC for Reducose^®^ 200 mg compared to placebo at 60 min (*p* < 0.001), 90 min (*p* = 0.013), 120 min (*p* = 0.024), 150 min (*p* = 0.01) and 180 min (*p* = 0.01). There was a significant reduction in the insulin iAUC for Reducose^®^ 225 mg dose compared to placebo at 60 min (*p* < 0.001), 90 min (*p* < 0.001), 120 min (*p* = 0.004), 150 min (*p* = 0.003), and 180 min (*p* = 0.013). Reducose^®^ 250 mg dose treatment also resulted in a significantly lower insulin iAUC compared to placebo at 60 min, 90 min, 120 min, 150 min, and 180 min (all *p* < 0.001). All three doses (200 mg, 225 mg and 250 mg) significantly lowered the iAUC 120 by 31%, 34% and 38%, respectively.

The peak plasma insulin values (Cmax) and the time of the plasma insulin peak (Tmax) for the three doses of Reducose^®^ and placebo are in Table 7. There was an overall significant difference in the peak insulin between the three doses of Reducose^®^ and placebo (χ^2^(3) = 28.427, *p <* 0.001). All three doses significantly lowered the peak insulin values (200 mg dose, *p* = 0.001; 225 mg and 250 mg doses, *p* < 0.001) compared to placebo. Considered together, the time to peak insulin was also significantly different between the three doses of Reducose^®^ and placebo (χ^2^(3) = 12.663, *p =* 0.005). However, post hoc pairwise analysis revealed that there was no change to the time to peak plasma insulin after the 200 mg dose and 250 mg dose of Reducose^®^ compared to placebo (*p* > 0.05), only the 225 mg (*p* = 0.013) dose significantly delayed the time to peak insulin.

There were no serious adverse events or adverse events reported during this study, and all doses of Reducose^®^ mulberry leaf extract were well tolerated by the participants.

## 4. Discussion

Previous research [22,23] has shown that Reducose^®^ white mulberry extract can reduce the postprandial blood glucose and plasma insulin responses to different pure carbohydrate tests. Lown et al. [22] showed a dose-dependent response to half (125 mg), normal (250 mg) and double (500 mg) doses of Reducose^®^ on lowering the blood glucose and blood insulin increase following a long chain carbohydrate challenge of 50 g maltodextrin. Thondre et al. [23] demonstrated that 250 mg Reducose^®,^ added directly into a 75 g sucrose challenge, positively affected its ability to reduce the blood glucose and blood insulin response. The study reported a 42% and 40% reduction in glucose iAUC 120 and insulin iAUC 120, respectively. These prior studies showed that Reducose^®^ is effective at limiting the blood glucose and insulin impact of carbohydrates with both α-1-4 glycosidic bonds and α-1-2 glycosidic bonds.

In this randomised, placebo-controlled trial, Reducose^®^, at the normal dose (250 mg) as well as lower dosages of 225 mg and 200 mg, was shown to significantly reduce the postprandial glucose response and postprandial plasma insulin response in normoglycemic individuals to a complete meal challenge including carbohydrates, proteins and fats. All three doses (200 mg, 225 mg, 250 mg) achieved the primary endpoint, significantly lowering iAUC 120 blood glucose by 30%, 33% and 32%, respectively, compared to placebo. Additionally, all three doses had a similar effect on lowering the iAUC 120 of plasma insulin by 31%, 34% and 38%, respectively. The median dose (225 mg) delayed the time of peak plasma insulin compared to placebo; however, between groups comparison of Tmax showed there were no significant differences between the dose groups. The results showed a clear and significant effect of Reducose^®^ to decrease both blood glucose and plasma insulin increases following a complete meal.

While prior studies on Reducose^®^ have demonstrated its effectiveness in reducing carbohydrate absorption and insulin response [22,23], these studies used a sugar or starch test meal. Although sugars and starches tend to represent the majority of the glycemic load in the Western diet, most of these are consumed in mixed meals, including proteins, fats and fibre. Proteins and amino acids have been shown to have an insulinotropic effect by direct β-cell stimulation [25], and high levels of free fatty acids from dietary fats can impair insulin sensitivity and lead to higher spikes in blood glucose levels [26]. Additionally, proteins [27] and fats [28] are known to delay gastric emptying, which has the potential to impact the results by delaying the release of the inhibitory compounds in Reducose^®^ from the stomach. These results demonstrate that Reducose^®^ is still able to significantly lower the GR and IR of mixed meals. The majority of this effect may occur through the inhibition of carbohydrate digestion; however, recent studies have shown that Reducose^®^ can also improve factors contributing to insulin resistance [29] through the promotion of IRS-1 phosphorylation. Reducose^®^ has also been shown to improve glucose stimulated insulin secretion through activation of the INS-2, PPAR-gamma and PDX-1 [30]. These additional effects on improved glucose disposal may help attenuate the hyperglycemic and insulinotropic effects of dietary fats and amino acids in the mixed meal challenge.

All three doses showed lower plasma insulin levels throughout the majority of the testing period until the late stage. For the 225 mg and 250 mg doses, 180 min following ingestion of the complete meal, the plasma insulin level remained higher following a drop-off in the placebo group. This late-stage increase in plasma insulin could be caused by the late-phase incretin response to incomplete carbohydrate digestion. Glucose-dependent insulinotropic peptide (GIP) and glucagon-like peptide-1 (GLP-1) play a large part in managing blood glucose levels following a meal and are primarily stimulated by carbohydrates in the intestine. GIP is produced by K-cells in the duodenum and jejunum, which are sensitive to glucose. GLP-1 is produced by L-cells, primarily in the latter end of the small intestine, jejunum and ileum, and are more sensitive to polysaccharides and disaccharides. Reducose^®^ is high in 1-deoxinojirimycin (DNJ), which is a potent α-glucosidase inhibitor and prevents a large number of disaccharides from being digested into glucose. This likely reduces the stimulation of K-cells in the early small intestine, leading to lower plasma insulin responses in the first 120 min following ingestion. However, as these undigested disaccharides pass through the small intestine, they continue to stimulate the L-cells, leading to increased secretion of GLP-1, which, among other actions, promotes glucose-stimulated insulin release. The glucose response curves show that between 120 min and 180 min, these dose groups still had elevated plasma glucose levels, so it is plausible that GLP-1 mediated insulin release is an explanatory factor for these higher than placebo insulin levels. This may also be a contributory factor to the small shift in time-to-peak insulin observed. This hypothesis is supported by a previous study which reported decreased early-stage GIP and increased late-phase GLP-1 release [31] and a similar finding of elevated late-phase insulin increase in a previous clinical study with Reducose^®^ [23].

The current study shows that Reducose^®^ mulberry leaf extract could be a useful tool for people trying to control their blood glucose and insulin responses, even at lower doses than previously reported [22,23]. Controlling postprandial glucose excursions is important for long-term health outcomes and reduces the complications in diseases such as Diabetes [5]. Glucose excursions are known to increase oxidative stress in microvascular/macrovascular tissue, and this risk is independent of diabetic status and continuous [6,7,8,9], and these risks increase substantially when postprandial glucose levels remain elevated above 7.8 mmol/L [32]. The results of this study showed that Reducose^®^ significantly lowered the postprandial glucose response, and even after eating a meal rich in carbohydrates, the peak postprandial glucose levels across all three dose groups were 5.7 mmol/L or lower.

There are several strengths to this paper. Most studies exploring the effects of mulberry leaf on postprandial glucose levels only utilise simple carbohydrates and do not evaluate the effects with a complete meal, and a larger meal. Many studies also fail to evaluate the effects on both glucose and insulin levels. Lowering glucose response when there is a corresponding increase in insulin levels places an unnecessary burden on the beta-cells from a food product and encourages the storage of excess glucose calories in fat reserves. A further strength of this study is that the dose relationship of Reducose^®^ was explored further, adding to the findings of Lown et al. [22].

This paper is not without limitations. It was important to evaluate the effects of Reducose^®^ on a complete meal, which at the time had not previously been explored or reported; however, the longer-term health benefits of continuous use of Reducose^®^ should be explored further. Future research directions should explore the longer-term metabolic health benefits, as well as investigate the effect of Reducose^®^ on the microbiome, as the undigested carbohydrates have been shown to reach the colon [33]; therefore, the effect on microbial composition and resultant impact on short-chain fatty acid production should be further explored.

## 5. Conclusions

The results of this study showed that the recommended 250 mg dose of Reducose^®^, as well as two lower doses significantly lowered both total and peak postprandial glycaemic and insulinaemic responses following a complete meal. The Reducose^®^ arms all had higher insulin levels compared with placebo 180-min after administration, indicating that the blood glucose moderating effects may in part be mediated by the stimulation of GLP-1. There are many health benefits to maintaining glucose and insulin levels within healthy ranges; however, achieving this often requires large lifestyle changes that can be difficult to maintain. Dietary supplements such as Reducose^®^ may provide a small habitual shift pre-meal to help make these changes easier and maintainable.

## Figures and Tables

**Table 1:** Physical characteristics of the recruited participants and those that completed all four study visits (mean ± SD).

	All Recruited Participants (*n* = 43)	All Completed Participants (*n* = 37)
Age (y)	29.4 ± 10.0	30.0 ± 10.4
Height (m)	1.7 ± 0.1	1.7 ± 0.1
Weight (kg)	67.8 ± 11.1	67.8 ± 10.6
BMI (kg/m^2^)	23.3 ± 2.5	23.2 ± 2.4
Fat mass (%)	24.4 ± 8.0	24.8 ± 8.0
Lean body mass (kg)	51.3 ± 10.2	50.9 ± 9.8

Abbreviations: kg = kilogram; m = metre; SD = standard deviation; y = years.

**Table 2:** Mean (±SD) blood glucose measurements (mmol/L) for Reducose^®^ 200 mg, 225 mg, 250 mg dietary supplement and placebo (*n* = 37).

Time (min)	Reducose^®^ 200 mg	Reducose^®^ 225 mg	Reducose^®^ 250 mg	Placebo
0	4.6 ± 0.3	4.6 ± 0.4	4.7 ± 0.4	4.7 ± 0.4
15	4.5 ± 0.4 *	4.4 ± 0.4 ^†^	4.5 ± 0.5	4.7 ± 0.5
30	5.2 ± 0.6 ^§^	5.1 ± 0.7 ^§^	5.2 ± 0.7 ^§^	5.9 ± 0.8
45	5.7 ± 0.8 ^†^	5.6 ± 0.7 ^§^	5.7 ± 0.7 ^§^	6.3 ± 0.9
60	5.6 ± 0.7	5.5 ± 0.7 *	5.6 ± 0.8	5.9 ± 0.9
90	5.1 ± 0.6	5.2 ± 0.7	5.2 ± 0.7	5.3 ± 0.7
120	5.1 ± 0.5	5.1 ± 0.7	5.2 ± 0.5	5.2 ± 0.8
150	5.1 ± 0.5	5.0 ± 0.6	5.1 ± 0.5	5.1 ± 0.6
180	4.9 ± 0.4	4.9 ± 0.6	4.9 ± 0.5	4.8 ± 0.6

Abbreviations: mg = milligrams; min = minutes; SD = standard deviation. Statistically significant difference compared with placebo: * = *p* < 0.05; ^†^ = *p* < 0.01; ^§^ = *p* < 0.001.

**Table 3:** Mean (±SD) glucose iAUC (mmol/L·min) at 60, 90, 120, 150 and 180 min for Reducose^®^ 200 mg, 225 mg and 250 mg dietary supplement and placebo (*n* = 37).

iAUC	Reducose^®^ 200 mg	Reducose^®^ 225 mg	Reducose^®^ 250 mg	Placebo
iAUC 60	32.6 ± 20.6 ^§^	27.8 ± 15.8 ^§^	30.4 ± 15.9 ^§^	54.6 ± 26.9
iAUC 90	55.7 ± 35.5 ^†^	50.6 ± 30.7 ^§^	53.1 ± 31.3 ^§^	83.3 ± 43.1
iAUC 120	71.7 ± 47.7 ^†^	68.5 ± 47.4 ^†^	69.7 ± 43.7 ^†^	102.5 ± 57.0
iAUC 150	87.2 ± 59.5 ^†^	83.7 ± 62.7 ^†^	84.5 ± 52.6 ^†^	118.8 ± 70.3
iAUC 180	99.3 ± 69.2 *	96.3 ± 78.3 *	95.5 ± 60.3 *	129.6 ± 79.4

Abbreviations: iAUC = incremental area under the curve; mg = milligrams; mmol/L·min = millimole per litre × minute; SD = standard deviation. Statistically significant difference compared with placebo: * = *p* < 0.05; ^†^ = *p* < 0.01; ^§^ = *p* < 0.001.

**Table 4:** Mean (±SD) peak blood glucose (mmol/L) and the time of the blood glucose peak (min) for Reducose^®^ 200 mg, 225 mg, 250 mg dietary supplement and placebo (*n* = 37).

	Reducose^®^ 200 mg	Reducose^®^ 225 mg	Reducose^®^ 250 mg	Placebo
Peak blood glucose	6.0 ± 0.6 ^†^	5.9 ± 0.7 ^§^	6.0 ± 0.7 ^†^	6.6 ± 0.8
Time of blood glucose peak	55.1 ± 24.8	61.2 ± 37.1	53.1 ± 27.1	47.4 ± 17.1

Abbreviations: mg = milligrams; mmol/L = millimole per litre; SD = standard deviation. Statistically significant difference compared with placebo: ^†^ = *p* < 0.01; ^§^ = *p* < 0.001.

**Table 5:** Mean (±SD) plasma insulin measurements (µU/mL) for Reducose^®^ 200 mg, 225 mg, 250 mg dietary supplement and placebo (*n* = 37).

Time (min)	Reducose^®^ 200 mg	Reducose^®^ 225 mg	Reducose^®^ 250 mg	Placebo
0	9.79 ± 4.94	9.30 ± 3.86	10.37 ± 3.83	10.36 ± 4.76
15	13.58 ± 8.81	12.38 ± 9.37 *	13.98 ± 6.52	18.18 ± 12.85
30	29.07 ± 17.34 ^§^	27.60 ± 20.46 ^§^	30.05 ± 14.59 ^†^	49.55 ± 29.20
45	45.25 ± 29.27 *	38.02 ± 18.11 ^§^	39.78 ± 19.56 ^§^	59.16 ± 34.43
60	43.07 ± 28.35	39.32 ± 20.42 *	36.20 ± 15.59 ^†^	53.20 ± 31.50
90	31.45 ± 16.96	31.96 ± 19.76	30.41 ± 17.83	40.11 ± 22.92
120	25.71 ± 11.70	26.69 ± 13.36	27.39 ± 13.36	32.82 ± 19.64
150	24.05 ± 13.01	22.93 ± 11.80	22.82 ± 9.84	26.18 ± 15.25
180	18.88 ± 10.67	20.23 ± 11.93 *	20.34 ± 10.61	16.63 ± 8.46

Abbreviations: mg = milligrams; min = minutes; SD = standard deviation; µU/mL = micro-international units per millilitre. Statistically significant difference compared with placebo: * = *p* < 0.05; ^†^ = *p* < 0.01; ^§^ = *p* < 0.001.

**Table 6:** Mean (±SD) plasma insulin iAUC (µU/mL·min) at 60, 90, 120, 150 and 180 min for Reducose^®^ 200 mg, 225 mg, 250 mg dietary supplement and placebo (*n* = 37).

iAUC	Reducose^®^ 200 mg	Reducose^®^ 225 mg	Reducose^®^ 250 mg	Placebo
iAUC 60	1136.35 ± 717.61 ^§^	1002.99 ± 639.23 ^§^	991.71 ± 532.46 ^§^	1763.05 ± 991.94
iAUC 90	1960.43 ± 1178.94 *	1769.64 ± 1089.45 ^§^	1679.62 ± 870.85 ^§^	2851.81 ± 1604.38
iAUC 120	2524.14 ± 1451.71 *	2393.81 ± 1445.54 ^†^	2235.56 ± 1194.78 ^§^	3634.91 ± 2089.11
iAUC 150	2977.11 ± 1673.58 *	2859.19 ± 1643.01 ^†^	2678.08 ± 1400.30 ^§^	4209.05 ± 2445.12
iAUC 180	3331.52 ± 1868.61 *	3224.65 ± 1824.11 *	3016.49 ± 1561.77 ^§^	4544.78 ± 2616.76

Abbreviations: iAUC = incremental area under the curve; mg = milligrams; µU/mL·min = micro-international units per millilitre × minutes; SD = standard deviation. Statistically significant difference compared with placebo: * = *p* < 0.05; ^†^ = *p* < 0.01; ^§^ = *p* < 0.001.

**Table 7:** Mean (±SD) peak plasma insulin (µU/mL) and the time of the plasma insulin peak (min) for Reducose^®^ 200 mg, 225 mg, 250 mg dietary supplement and placebo (*n* = 37).

	Reducose^®^ 200 mg	Reducose^®^ 225 mg	Reducose^®^ 250 mg	Placebo
Peak plasma insulin	52.62 ± 31.52 ^§^	45.74 ± 21.62 ^§^	45.51 ± 17.55 ^§^	68.69 ± 33.35
Time of plasma insulin peak	54.73 ± 26.74	63.24 ± 31.05 *	56.76 ± 26.49	44.59 ± 13.46

Abbreviations: mg = milligrams µU/mL = micro-international units per millilitre; SD = standard deviation. Statistically significant difference compared with placebo: * = *p* < 0.05; ^§^ = *p* < 0.001.

## Data Availability

The data presented in this study are available on request from the corresponding author. The data are not publicly available due to intellectual property rights.
